# Return to Sport Following Arthroscopic Management of Femoroacetabular Impingement: A Systematic Review

**DOI:** 10.3390/jcm13175219

**Published:** 2024-09-03

**Authors:** Ludovico Lucenti, Nicola Maffulli, Tommaso Bardazzi, Raoul Saggini, Michael Memminger, Francesco Simeone, Filippo Migliorini

**Affiliations:** 1 Department of Precision Medicine in Medical, Surgical and Critical Care (Me.Pre.C.C.), University of Palermo, 90133 Palermo, Italy; ludovico.lucenti@gmail.com; 2 Department of Trauma and Orthopaedic Surgery, Faculty of Medicine and Psychology, University La Sapienza, 00185 Rome, Italy; n.maffulli@qmul.ac.uk; 3 School of Pharmacy and Bioengineering, Faculty of Medicine, Keele University, Stoke on Trent ST4 7QB, UK; 4 Centre for Sports and Exercise Medicine, Barts and the London School of Medicine and Dentistry, Mile End Hospital, Queen Mary University of London, London E1 4DG, UK; 5 Department of Orthopaedic and Trauma Surgery, Academic Hospital of Bolzano (SABES-ASDAA), 39100 Bolzano, Italy; tommaso.bardazzi@sabes.it (T.B.); michael.memminger@sabes.it (M.M.); francesco.simeone@sabes.it (F.S.); 6 Department of Life Sciences, Health and Health Professions, Link Campus University, 00165 Rome, Italy; raoul.saggini@gmail.com

**Keywords:** femoroacetabular impingement, FAI, return to sport, arthroscopy, orthopaedics

## Abstract

** Background:** Femoroacetabular impingement (FAI) is common. The present systematic review updates the current evidence on return to sport (RTS) in patients who have undergone arthroscopic surgery for FAI in any of its variants (CAM, pincer, or both). **Methods:** The outcomes of interest were sports-related patient-reported outcome measures (PROMs) and the level and time to RTS. All available clinical studies concerning the RTS following arthroscopic management of FAI were considered. In July 2024, the following databases were accessed following the PRISMA guidelines: Embase, Web of Science, and PubMed. Only studies with a minimum of six months of follow-up were eligible. **Results:** From 1245 initially identified articles, 43 studies (4103 patients) met the inclusion criteria, in which 32.1% (1317 of 4103 patients) were women. The mean length of follow-up was 33.7 ± 15.8 months. The mean age was 28.1 ± 7.2 years, the mean BMI was 24.7 ± 6.4 kg/m^2^, and 79.6% ± 27.8% of patients returned to sport at the same or higher level at a mean of 14.3 ± 9.6 months. The mean time away from sports was 8.0 ± 3.3 months. **Conclusion:** Arthroscopic management for FAI leads to a high rate of RTS, with approximately 80% of patients returning to their preinjury level. Future research should focus on standardised definitions of RTS, sport-specific rehabilitation protocols, and the influence of deformity and procedures on RTS.

## 1. Introduction

Femoroacetabular impingement (FAI) is common, with an incidence of 30–70 per 100,000 individuals per year [1,2,3]. A mechanical conflict between the femoral head and the acetabulum causes this condition. Three main pathoanatomical morphologies characterise FAI. In cam morphology, there is a loss of sphericity of the femoral head, which is three times more common in male athletes. The pincer morphology is an overcoverage of the acetabulum and is more common in women. In mixed morphology, the most common deformity is evident in both cam and pincer morphologies [4,5]. FAI is a common finding on imaging [6,7]. FAI is often of uncertain clinical significance, asymptomatic, and reported as an occasional finding on plain radiographs. Approximately 40% of patients with hip disorders have evidence of FAI at imaging [8]. In patients with soft tissue damage, articular damage or several mechanical symptoms, FAI might become symptomatic. If left untreated, FAI might lead to labral tears and chondral damage. These patients develop groin pain, disability, limited range of motion (ROM), and mechanical symptoms such as clicking or catching in the hip [9,10]. FAI is more common in contact sports involving recurrent hip flexion and rotation, such as hockey, soccer, basketball, and martial arts [11,12]. FAI remarkably reduces physical performance in some athletes, forcing them to leave the playing field [12,13,14,15]. In selected athletes with persistent symptoms of FAI, arthroscopy might be recommended to restore bony anatomy and manage associated labral and chondral lesions. The efficacy of arthroscopy in athletes has been documented; however, the time and the level of the return to sport (RTS) after arthroscopic management remains a critical area of investigation [16,17]. Evidence on FAI is rapidly evolving, attracting broad research and innovations. The published literature is growing exponentially; however, the rate, timing, and success of RTS after hip arthroscopy for FAI in athletes are still controversial [10]. Therefore, a systematic review was conducted. The present investigation aims to update current evidence on RTS in patients who have undergone arthroscopic surgery for FAI, discussing possible influencing factors and new prospects. The outcomes of interest were sports-related patient-reported outcome measures (PROMs) and the level and time to RTS.

## 2. Methods

### 2.1. Eligibility Criteria

All available clinical studies concerning the arthroscopic management of FAI were considered. All types of FAI were considered in the present investigation. Only articles published in peer-reviewed journals were included. According to the authors’ language capabilities, English, Spanish, French, Italian, or German articles were eligible. Only studies classified as evidence levels I to III according to the 2020 Oxford Centre of Evidence-Based Medicine [18] were considered. Editorials, opinions, reviews, and letters were excluded, as well as animal studies, computational analyses, in vitro experiments, cadaveric research, or biomechanical assessments. Studies that considered open surgery were excluded, as were studies with less than six months of follow-up. Finally, only articles focusing on RTS were included in the present review.

### 2.2. Search Strategy

The current systematic review followed the guidelines outlined in the Preferred Reporting Items for Systematic Reviews and Meta-Analyses (PRISMA) statement of 2020 [19]. The PRISMA 2020 checklist was used (see Supplementary Materials) [19]. There was not a systematic review registration of the present study. The following algorithm was implemented for the literature search:

- Problem: FAI;
- Intervention: arthroscopic management;
- Design: clinical trial;
- Outcome: return to sports;
- Follow-up: minimum of 6 months.

In July 2024, the following databases were accessed: Embase, Web of Science, and PubMed, with no additional filters or time constraints. The Medical Subject Headings (MeSH) used for the database were: “femoroacetabular impingement/complications” [MeSH] OR “femoroacetabular impingement/diagnosis” [MeSH] OR “femoroacetabular impingement/etiology” [MeSH] OR “femoroacetabular impingement/physiopathology”[MeSH] OR “fibrocartilage/physiopathology” [MeSH] OR “hip” [MeSH] OR “hip/physiopathology” [MeSH] OR “hip joint/pathology” [MeSH] OR “pain” [MeSH] OR acetabulum OR cam OR cam impingement OR cam type OR FAI OR FAIS OR femoroacetabular impingement OR hip OR hip injury OR ice hockey injury OR pincer impingement OR pincer type OR sports injury) AND (“athletes” [MeSH] OR “athletic injuries/physiopathology” [MeSH] OR “athletic injuries/surgery”[MeSH] OR “dancing” [MeSH] OR “football/injuries” [MeSH] OR “hockey/injuries” [MeSH] OR “soccer/injuries” [MeSH] OR “tennis” [MeSH] OR athletes OR athletic performance OR elite athletes OR female athlete OR ice hockey player OR soccer OR soccer players OR sport type OR sports OR tennis) AND (“acetabulum/surgery” [MeSH] OR “arthralgia/surgery” [MeSH] OR “Arthroscopy” [MeSH] OR “Arthroscopy/methods” [MeSH] OR “cartilage, articular/surgery” [MeSH] OR “femoroacetabular impingement/surgery” [MeSH] OR “fibrocartilage/surgery” [MeSH] OR “hip injuries/surgery” [MeSH] OR “hip joint/surgery” [MeSH] OR “joint capsule/surgery” [MeSH] OR “joint diseases/surgery” [MeSH] OR arthroscopic surgical procedures OR arthroscopy OR hip arthroscopic surgery OR hip arthroscopy) AND (“patient reported outcome measures” [MeSH] OR “patient satisfaction” [MeSH] OR “return to sport” [MeSH] OR “return to sport/statistics & numerical data” [MeSH] OR “treatment outcome” [MeSH] OR “visual analog scale” [MeSH] OR outcomes OR return to play OR return to soccer OR return to sport OR return to sports OR return-to-running OR return-to-sport.

### 2.3. Selection and Data Collection

Two authors (F.S. and T.B.) performed the database search. All retrieved titles underwent manual screening, and their abstracts were accessed if deemed appropriate. Full texts were examined in cases where there was a match. Articles without accessible full texts were excluded from consideration. A cross-reference of the bibliographies of full-text articles was also conducted for potential inclusion. A third author (N.M.), who made the final decision, resolved any disagreements among authors.

### 2.4. Data Items

Two authors (F.S. and T.B.) performed data extraction. The following data at baseline were extracted: author, year of publication and journal, length of the follow-up, number of patients with related mean age, and BMI. Data on the following PROMs at baseline and at the last follow-up were retrieved: University of California, Los Angeles (UCLA) activity score [20], Hip Outcome Score—Sport-Specific Subscale (HOS-SSS) [21], subscales function in sports and recreation and participation in physical activities of the Copenhagen Hip and Groin Outcome Score (HAGOS) [22], and Tegner Score [23]. Moreover, information on the level and time to RTS was collected. Data were extracted in Microsoft Office Excel version 16.0 (Microsoft Corporation, Redmond, WA, USA).

### 2.5. Assessment of Risk of Bias

The assessment of risk of bias (RoB) followed the guidelines outlined in the *Cochrane Handbook for Systematic Reviews of Interventions* [24]. Two authors (F.S. and T.B.) independently evaluated the RoB in the extracted studies. Since only nonrandomised controlled trials (non-RCTs) were included in this review, the Risk of Bias in Nonrandomized Studies of Interventions (ROBINS-I) tool [25] was employed. This tool assesses seven domains of potential bias in non-RCTs, including domains focusing on possible confounding factors and patient selection characteristics before the comparative intervention, bias in classification during the intervention, as well as methodological quality post-intervention comparison, which involves deviations from previously intended interventions, missing data, erroneous outcome measurement, and bias in reported outcome selection. The ROBINS-I chart was generated using Robvis software (Riskofbias.info, Bristol, UK) [26].

### 2.6. Synthesis Method

The main author (F.M.) performed the statistical analyses following the recommendations of the *Cochrane Handbook for Systematic Reviews of Interventions* [24]. IBM SPSS software version 25 was used. The arithmetic mean and standard deviation were used for continuous data, and the frequency (events/observations) for dichotomic variables. The mean difference effect measure and unpaired t-test were used to evaluate sports-related PROM improvement from baseline to the last follow-up. Values of *p* < 0.05 were considered statistically significant.

## 3. Results

### 3.1. Study Selection

**Figure 1:**
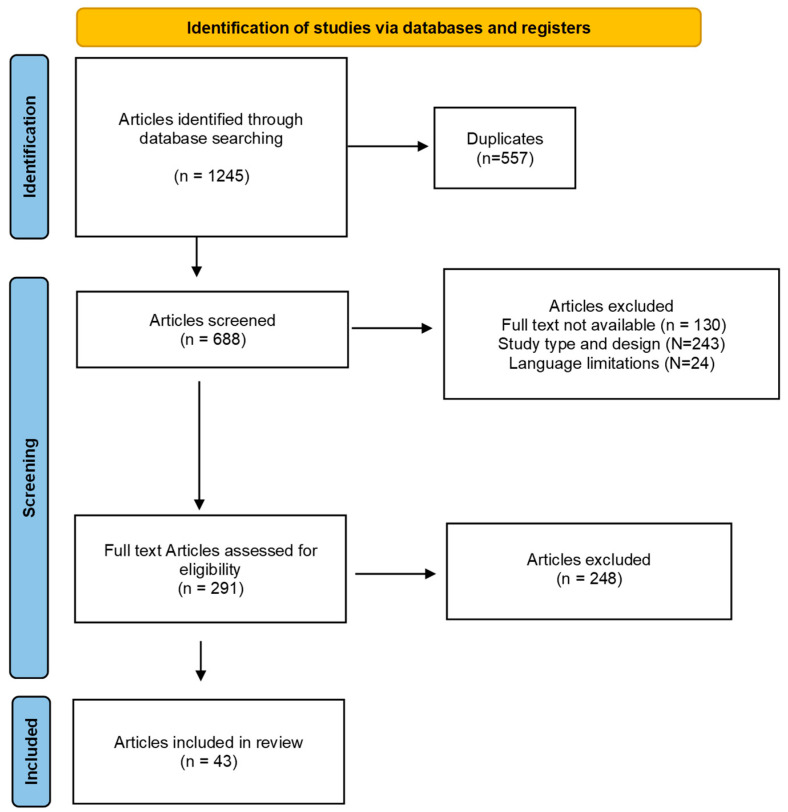
PRISMA flow chart of the literature search.

Our initial search uncovered 1245 articles potentially relevant to the research question. After eliminating duplicates, we assessed the eligibility of 688 articles based on their abstracts. Overall, 397 articles failed to meet the inclusion criteria due to several factors. The primary reason for exclusion was a mismatch with the predetermined study design (*n* = 243). Additionally, limitations in full-text accessibility (*n* = 130) and language barriers (*n* = 24) resulted in further exclusions. Following a meticulous full-text review of the remaining 291 articles, an additional 246 were excluded. This stringent selection process yielded a final selection of 43 studies for inclusion in this systematic review. The results of the literature search are shown in Figure 1.

### 3.2. Risk-of-Bias Assessment

**Figure 2:**
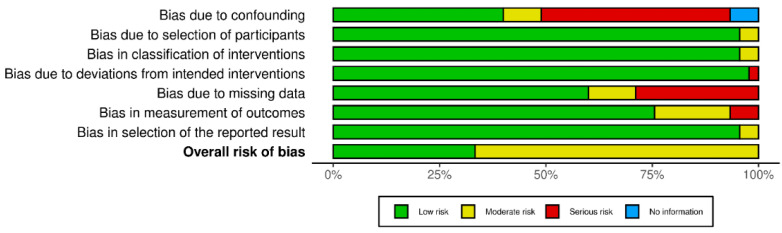
ROBINS-I levels of non-RCTs.

Due to the absence of randomised controlled trials (RCTs) in the included studies, the risk of bias was assessed using the ROBINS-I tool. A concerning finding emerged in the first domain, where nearly two-thirds of the studies exhibited a serious or moderate risk of bias due to confounding. This highlights a potential limitation in the overall methodological quality of the studies. Encouragingly, the risk of bias from participant selection was generally low across all studies. Furthermore, a low risk of bias was maintained in classifying interventions and adherence to the intended intervention protocol for nearly all studies. However, the domains assessing post-intervention bias revealed concerns regarding outcome measurement and missing data. The selection of reported results presented minimal concerns in almost all studies. In conclusion, the ROBINS-I assessment indicated a moderate or low overall risk of bias across the included studies, suggesting an acceptable level of methodological quality, albeit with a caveat regarding confounding (Figure 2).

### 3.3. Study Characteristics and Results of Individual Studies

Data from 3964 patients were retrieved. Of them, 39.4% (1276 of 3235 patients) were women. The mean length of follow-up was 34.0 ± 16.0 months. The mean age was 28.1 ± 7.3 years, and the mean BMI was 24.7 ± 6.5 kg/m^2^. Generalities of the included studies are shown in Table 1.

### 3.4. Results Synthesis

79.6 ± 27.8% of patients returned to sport at the same or higher level at a mean of 14.3 ± 9.6 months. The mean time away from sports was 8.0 ± 3.3 months. A significant improvement was found in HOS-SSS, but no difference was found in the other sports-related PROMs (Table 2).

## 4. Discussion

FAI is a common cause of groin pain, especially among young individuals and athletes [10,38,70]. The literature reports optimal results after arthroscopic management of FAI. According to the main findings of the present systematic review, most patients returned to sport after hip arthroscopy for FAI. Indeed, at a mean of 14.3 months, approximately 80% of patients returned to sport at the same or higher level. The rate of RTS after arthroscopy for FAI is high, especially compared to conservative management. Poor evidence reports that following conservative management, approximately 50% of patients did not return to their previous sport [71,72]. Therefore, given the high rate of RTS, arthroscopic management for FAI spread. In a previous systematic review of 10 studies (376 patients), Annin et al. [73] reported that 74.2% to 100% of athletes returned to the sport at the same or greater level compared with the preinjury level, and 0% to 25.8% returned to a lower level. The collective mean follow-up reported by the authors ranged from 24 to 240 months, similar to that reported in the present systematic review. A previous systematic review of 31 articles and 1911 patients found that 87.7% returned to sports [74], while another systematic review of 35 studies demonstrated a 91% RTS four to ten months after arthroscopic FAI [75]. A systematic review of 15 studies (809 patients) showed that 88.3% returned to play and 85.5% returned to play at the preinjury level after arthroscopy for FAI [76].

The present systematic review did not evidence a statistically significant improvement in PROMs from baseline to the last follow-up, except in HOS-SSS, which improved from 43.2 to 76.5. However, although not statistically significant, PROMs improved from baseline to the last follow-up. The mean UCLA Activity Score increased slightly from 7.7 to 9.4, indicating increased activity levels. Similarly, the mean Tegner Activity Scale increased from 4.5 to 5.2. The subscales of function in sports and recreation and participation in physical activities of the HAGOS score also improved from baseline to the last follow-up. PROMs are largely used in the literature to evaluate surgery outcomes [77]. Conversely, previous studies showed significant improvement in PROMs in the present investigation. Domb et al. [78] reported a significant improvement in PROMs, sports participation, and patient satisfaction in 177 athletes at a mean of 10 years of follow-up. Another clinical investigation on 4963 patients who underwent arthroscopy for FAI [79] reported a statistically significant improvement in PROMs at 12 months follow-up. The authors hypothesised that heterogeneities in sports, pathoanatomical deformities, soft tissue lesions, and management protocols might have improved data variability, leading to non-significant results. This statement is supported by the higher values of standard deviations observed in some PROMs, especially at baseline. Pathoanatomical deformities and associated lesions likely vary significantly in their impact and progression, further contributing to the complexity of the data. Heterogeneities in sports types can lead to various physical demands and activity levels, each interacting differently with specific anatomical and pathological conditions. A previous clinical investigation categorised sports in contact, noncontact pivoting, and noncontact nonpivoting on 189 young athletes [80]. A total of 81 athletes (42.9%) failed to return to preinjury sports at their preinjury level. In comparison, 108 athletes (57.1%) returned to sport at their preinjury level. These values are considerably lower than those reported in the literature, probably due to the authors’ strict definitions and categorisation of RTS. Different types and classifications of sports have been considered and compared in RTS following arthroscopy for FAI. Weber et al. [69], in a study of 49 hip arthroscopies on 39 athletes, compared cutting, contact, impingement, asymmetric/overhead, and endurance athletes, with no difference in RTS. A similar classification but with contrasting results was used in another systematic review of 29 articles (1426 hip arthroscopies) [81]. Flexibility athletes had the highest rate of RTS after hip arthroscopy (94.8%), while contact athletes had the lowest rate (88%) [81]. Endurance athletes had the faster RTS (5.4 ± 2.6 months) [81]. Other authors classified different types of sports as high-impact and low-impact [82], while others focused their research only on a single sport [83]. The heterogeneity in the types of sports may lead to variability in outcomes: athletes involved in high-impact sports such as soccer and hockey tend to have different recovery trajectories compared to those in low-impact sports such as swimming. This variability also highlights the need for sport-specific rehabilitation protocols to optimise RTS outcomes [84].

Differences in RTS among the included studies can arise from a difference in the definition of RTS [85]. Indeed, RTS can be considered a continuum of three elements: return to participation, return to sport proper, and return to performance. In return for participation, athletes may join in rehabilitation, training, or sport at a lower intensity than their RTS goal. During RTS, athletes return to specific sports without performing at their desired routine levels. Return to performance is an expansion of RTS: the athlete has gradually returned to his sport and is performing at or above his preinjury level [86].

The type of surgical intervention influences the outcomes and the RTS. Labral debridement, repair, and reconstruction are three different management modalities of FAI. Labral debridement requires cutting and levelling areas of torn or damaged labrum. In contrast, labral repair uses anchors and sutures to bundle tissue together and reattach the labrum to its anatomic position [87,88]. The damaged labrum is removed and replaced with an autologous, heterologous, or synthetic graft using various reconstruction techniques [89,90]. The type of surgical procedure influences the time and rate of RTS. Scanaliato et al. [63], in a study on labral reconstruction for FAI in 30 athletes, reported that 86.7% (26 of 30 patients) returned to the sport in a mean time of 6.6 months. Another investigation, which included 32 athletes who underwent primary arthroscopic labral reconstruction for FAI, reported an RTS of 78% at a mean follow-up of 26.4 months [91]. Mohan et al. [46] conducted a study with a mean follow-up of 34 months on 50 amateur athletes who have undergone labral repair. The authors reported a rate of RTS of 92% (46/50). In another clinical investigation [45] evaluating mid-term outcomes after hip arthroscopy and labral debridement for cam-type FAI, all 108 analysed patients returned to sport with no limitation at a mean of 2.6 months. In the present investigation, the use of these surgical techniques varied largely among studies. Debridement and repair were the most commonly used modalities (22 of 45 articles). Labral repair was performed in 13 investigations. Debridement, repair, and reconstruction were used in nine studies. Only two articles performed labral reconstruction, one reconstruction associated with repair, and one study reported data on isolated debridement.

The findings of the present review underscore the importance of considering individual patient factors, such as the type of FAI, the chosen labral procedure, the patient’s level of athletic activity, and the type of sport when planning arthroscopic management and establishing the possible RTS.

The present systematic review has limitations. We did not include languages other than English, Spanish, French, Italian, or German. Studies with levels of evidence IV and V were not included to increase the quality of recommendations. The included studies considered various types of FAI (cam, pincer, and mixed), each with peculiar anatomical and biomechanical characteristics, treatment responses, and variability in RTS and PROMs. The morphology of the FAI can influence the outcomes and RTS. Most studies did not consider the morphology of the FAI separately in terms of timing, outcomes, and results on RTS after hip arthroscopy for FAI [17]. Other limitations are evident. The predominance of retrospective investigations increases the risk of selection bias. Many included studies had varied patient populations, ranging from high-level athletes to recreational sports participants, so the level of pre-injury athletic activity, age, and BMI varied significantly across studies. All these factors can influence the results [92,93,94,95,96]. Additionally, there is variability regarding the types of sports, and the different activity levels complicate the interpretation of the overall RTS [41,97,98,99]. Given the lack of information in most studies, it was not possible to analyse the different levels of leagues separately. Physical rehabilitation and psychological factors such as an athlete’s motivation, confidence in the surgical repair, and fear of re-injury can significantly affect RTS outcomes. Future studies should overcome current limitations, evaluating individual factors, such as the type of FAI, the chosen labral procedure, the level of athletic activity, and the type of sport separately.

## 5. Conclusions

The literature reports optimal results after arthroscopic management of FAI. According to the main findings of the present systematic review, most patients returned to sport after hip arthroscopy for FAI. Indeed, at a mean of 14.3 months, approximately 80% of patients returned to sport at the same or a higher level.

## Figures and Tables

**Table 1:** Generalities of the included studies.

Author and Year	Country	Journal	Design	Follow-Up (months)	Labral Procedure	Patients (*n*)	Women (*n*)	Mean Age	Mean BMI
Abrahamson et al., 2020 [27]	Sweden	J Exp Orthop	Prospective	23.4	Debridement	135	127	30.0	
						416			
Botser et al., 2014 [28]	USA	Am J Orthop	Prospective	14.3	Repair	18	23	20.1	
Charles et al., 2021 [29]	Belgium	PLoS One	Retrospective	53.0	Debridement and Repair	54	25	35.0	
Ferrer-Rivero et al., 2024 [30]	Spain	J ISAKOS	Retrospective	32.4		11	11	32.0	21.7
						22	22		
Frank et al., 2019 [31]	USA	Orthop J Sports Med	Retrospective	31.2	Repair	97	97	36.0	23.8
						97	97	37.8	27.4
Glaws et al., 2019 [32]	USA	J Sport Rehabil	Prospective	6.0	Repair	16	7	23.4	24.2
						12	8	27.4	25.4
Hassebrock et al., 2020 [33]	USA	Am J Sports Med	Retrospective	26.0	Repair	22	6	22.0	26.7
						28	12	19.0	25.4
Hassebrock et al. 1, 2020 [34]	USA	Am J Sports Med	Retrospective	24.0	Repair	49	17	19.4	23.2
						62	18	18.6	24.6
Larson et al., 2019 [35]	USA	Arthroscopy	Retrospective	39.8	Repair	28	7	15.9	
Levy et al., 2016 [36]	USA	Am J Sports Med	Retrospective	24.0	Debridement and Repair	51	29	26.3	23.7
Lindman et al., 2020 [37]	Sweden	Am J Sports Med	Prospective	60.0	Debridement and Repair	64	12	24.0	23.7
Lindman et al., 2021 [38]	Sweden	Orthop J Sports Med	Prospective	24.0		172	3	28.0	25.6
Litrenta et al., 2020 [39]	USA	J Pediatr Orthop	Retrospective	45.2	Debridement and Repair and Reconstruction	69		15.9	21.4
Maldonado et al., 2020 [40]	USA	BMC Musculoskelet Disord	Retrospective	66.8	Debridement and Repair and Reconstruction	25	14	41.4	25.1
Marom et al., 2023 [41]	USA	Knee Surg Sports Traumatol Arthrosc	Retrospective	72.0	Debridement and Repair	119	51	21.6	23.7
Martinez et al., 2020 [42]	Spain	Arthrosc Sports Med Rehabil	Retrospective	24.0		76	22	38.8	29.0
						50	2	33.7	4.0
						36	22	36.1	61.2
						18	3	36.8	16.7
Martinot et al., 2020 [43]	France	Orthop Traumatol Surg Res	Retrospective	55.2	Debridement and Repair	55	14	32.3	24.7
McConkey et al., 2019 [44]	USA	J Pediatr Orthop	Prospective	24.0		12	5	15.7	20.3
						12	5	16.5	21.5
Migliorini et al., 2023 [45]	Italy	J Orthop Surg Res		72.8	Debridement	108	46	41.5	27.0
Mohan et al., 2017 [46]	USA	Arthroscopy	Retrospective	34.0	Debridement and Repair and Reconstruction	50	33	17.8	23.1
Mullins et al., 2019 [47]	Ireland	Knee Surg Sports Traumatol Arthrosc	Prospective	12.0	Repair	47		24.6	
						32		24.3	
Mullins et al., 2021 [48]	Ireland	Orthop J Sports Med	Prospective	27.1	Repair	760	50	26.3	
Nho et al., 2011 [49]	USA	Am J Sports Med	Retrospective	27.0	Debridement and Repair	47	13	22.8	
						47	13	22.8	
Ortiz-Declet et al., 2019 [50]	USA	Arthroscopy	Retrospective	47.4	Debridement and Repair and Reconstruction	34	19	20.8	22.8
Owens et al., 2022 [51]	USA	Orthop J Sports Med	Prospective	36.1	Debridement and Repair and Reconstruction	30	16	29.4	25.9
				34.5		60	33	27.5	24.8
Owens et al., 2022 [52]	USA	Orthop J Sports Med	Retrospective	30.2	Reconstruction	29		40.3	27.8
				27.6		29	29	40.5	27.1
Palmer et al., 2012 [53]	USA	Arthroscopy	Retrospective	46.0	Repair	185	102	40.2	
Parvaresh et al., 2021 [54]	USA	Phys Ther Sport	Retrospective	24.0	Repair	23	14	36.2	22.8
Perets et al., 2018 [55]	USA	Arthroscopy	Retrospective	49.1	Debridement and Repair and Reconstruction	60	46	19.5	22.4
Philippon et al., 2007 [56]	USA	Knee Surg Sports Traumatol Arthrosc	Retrospective	19.2	Debridement and Repair and Reconstruction	45	3	31.0	
Philippon et al., 2010 [57]	USA	Am J Sports Med	Retrospective	24.0	Repair	28		27.0	
Polesello et al., 2014 [58]	Brazil	Hip Int	Retrospective	73.2	Debridement and Repair	24	3	34.6	
Ramos et al., 2020 [59]	USA	J Hip Preserv Surg	Retrospective	19.2	Repair	10		45.0	
Rosinsky et al., 2019 [60]	USA	Am J Sports Med	Retrospective	35.0	Debridement and Repair and Reconstruction	44	19	17.3	23.3
						38	30		23.7
Saito et al., 2019 [61]	Japan	Am J Sports Med	Retrospective	24.0	Repair and Reconstruction	25		17.5	22.2
								22.0	22.4
								18.5	22.0
								17.0	22.4
Sansone et al., 2015 [62]	Sweden	Orthop J Sports Med	Retrospective	12.3	Debridement and Repair	85	21	25.0	
Scanaliato et al., 2020 [63]	USA	Arthroscopy	Retrospective	24.3	Reconstruction	30	17	30.4	23.4
Singh et al., 2010 [64]	Australia	Arthroscopy	Prospective	22.0	Repair	24		22.0	24.0
Snaebjörnsson et al., 2022 [65]	Sweden	Arthroscopy	Prospective	24.0	Debridement and Repair	84	17	19.8	
Tjong et al., 2016 [66]	USA	Orthop J Sports Med	Retrospective	24.0		13	7	44.0	
						10	8	43.7	
Tran et al., 2013 [67]	Australia	ANZ J Surg	Retrospective	14.0	Debridement and Repair	34	5	15.7	
Ukwuani et al., 2018 [68]	USA	Arthroscopy	Retrospective	23.0	Debridement and Repair	21	62	19.9	21.9
						43		23.3	23.2
Weber et al., 2020 [69]	USA	Orthop J Sports Med	Retrospective		Debridement and Repair	1	2	19.5	
						15	2		
						8	1		
						6			
						9	6		

**Table 2:** Results of PROMs (MD: mean difference; FU: follow-up; PROMs: patient-reported outcome measures; UCLA: University of California, Los Angeles; HOS-SSS: Hip Outcome Score—Sport-Specific Subscale; HAGOS: Copenhagen Hip and Groin Outcome Score).

PROMs	At Baseline	At Last FU	MD	*p*
UCLA activity score	7.7 ± 3.9	9.4 ± 0.7	1.7	0.4
HOS-SSS	43.2 ± 21.8	76.5 ± 23.8	33.2	<0.01
Tegner score	4.5 ± 2.0	5.2 ± 2.4	0.7	0.1
HAGOS Function in sports and recreation	64.8 ± 23.7	72.8 ± 8.3	8.1	0.09
HAGOS Participation in physical activities	59.7 ± 20.1	66.4 ± 12.9	6.7	0.08

## Supplementary Materials

The following supporting information can be downloaded at: https://www.mdpi.com/article/10.3390/jcm13175219/s1, The PRISMA 2020 checklist.
